# EVERYDAY DISCRIMINATION AND RACIAL/ETHNIC DISPARITIES IN COGNITIVE FUNCTIONING

**DOI:** 10.1093/geroni/igad104.0182

**Published:** 2023-12-21

**Authors:** Wen-Hua “Zoey” Lai

**Affiliations:** Michigan State University, East Lansing, Michigan, United States

## Abstract

Alzheimer’s disease and related dementias are expected to triple from 5 million to 13.7 million by 2050 in the U.S. Black, and Latinx older adults experience poorer cognitive health and a 1.5 to 2 times higher rate of dementia than their white counterparts. Due to the structural racism in the U.S., chronic exposure to everyday discrimination may contribute to cognitive health inequalities, yet limited empirical research examines this association. This study uses parallel linear growth curve modeling to assess the impact of long-term exposure to everyday discrimination on the initial level of cognition (i.e., latent intercept) and change in cognition (i.e., latent slope) among older adults and examines whether such association varies among Black, U.S.-born Latinx, foreign-born Latinx, and white older adults. Using data from the 2006-2012 Health and Retirement Study (N=8,195), these findings show that racial/ethnic minority older adults, especially Blacks, experience greater everyday discrimination and poorer cognitive health than their white peers. Greater exposure to everyday discrimination was associated with lower initial levels of cognitive function and a faster decline in cognitive function. Results from multiple groups analysis further indicate that everyday discrimination shaped cognitive trajectories only for white and Black older adults but not for U.S. -born and foreign-born Latinx older adults. These findings suggest that white and Blacks older adults who experience a higher level of everyday discrimination may be vulnerable to cognitive decline. This study discusses the implication of these unexpected findings and future research directions.

